# Programmed death ligand‐1 expression in gastrointestinal cancer: Clinical significance and future challenges

**DOI:** 10.1002/ags3.12348

**Published:** 2020-06-11

**Authors:** Kohei Yamashita, Masaaki Iwatsuki, Jaffer A. Ajani, Hideo Baba

**Affiliations:** ^1^ Department of Gastroenterological Surgery Graduate School of Medical Sciences Kumamoto University Kumamoto Japan; ^2^ Department of Gastrointestinal Medical Oncology The University of Texas MD Anderson Cancer Center Houston TX USA

**Keywords:** gastrointestinal cancer, heterogeneity, PD‐L1, predictive biomarker

## Abstract

Cancer immunotherapy has caused a paradigm shift from conventional therapies that directly target cancer cells to innovative therapies that utilize the host immune system. In particular, programmed cell death‐1 (PD‐1)/programmed death ligand‐1 (PD‐L1) inhibitors have achieved an impressive breakthrough and been approved for clinical use in several types of cancer including gastrointestinal (GI) cancer. To identify and develop predictive biomarkers for PD‐1 inhibitors is of great concern in clinical practice. Although PD‐L1 expression is considered a logical biomarker as PD‐L1 is a substantial target of the immune checkpoint inhibitors, its clinical significance in GI cancer remains unclear. In this review, we summarize the current evidence for PD‐L1 expression as a prognostic and predictive biomarker for PD‐1/PD‐L1 inhibitors in GI cancer from recent publications, and emerging evidence from recent key clinical trials on the efficacy of PD‐1/PD‐L1 inhibitors. Challenging clinical issues for PD‐L1 assessment are then discussed from the viewpoint of the methodology for PD‐L1 evaluation including the differences in PD‐L1 detection assays and evaluation criteria for PD‐L1 positivity. Moreover, we highlight the biological features of PD‐L1 expression in terms of tumor spatial and temporal heterogeneity, which suggests important implications for biomarker analysis. Finally, we describe future perspectives using liquid biopsy for better assessment of PD‐L1 status. This new information should improve our understanding of the clinical significance of PD‐L1 in GI cancer, leading to optimal patient selection and treatment strategy for the clinical use of PD‐1/PD‐L1 inhibitors in patients with GI cancer.

## Graphical abstract

PD‐L1 expression is considered a promising predictive biomarker for PD‐1 inhibitors in gastrointestinal cancer. Considering challenging issues in assessing PD‐L1 expression will have important implications for better understanding the significance of PD‐L1 expression as a biomarker.

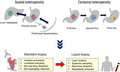

## 1 INTRODUCTION

The concept of cancer immunity has given rise to new insights into oncology.^1^ Notably, cancer immunotherapy has caused a paradigm shift from conventional therapies that directly target cancer cells to innovative therapies that utilize the host immune system.^2^ Immune checkpoint inhibitors (ICIs), which target inhibitory receptors on immune effector cells and reactivate the immune response, have been highlighted over the past several years.^3^ The programmed cell death‐1 (PD‐1)/programmed death ligand‐1 (PD‐L1) axis has been attracting particular interest as a promising target for ICIs since it was first described in 1992.^4^ Subsequently, blockade of the PD‐1/PD‐L1 axis has demonstrated favorable antitumor effects and achieved an impressive breakthrough in cancer immune therapy for several types of cancer including melanoma, non‐small cell lung cancer (NSCLC), and gastrointestinal (GI) cancer.^5^, ^6^, ^7^, ^8^

To identify and develop predictive markers for ICIs is of great concern in clinical practice. To date, several predictive markers for PD‐1/PD‐L1 inhibitors, such as tumor mutation burden (TMB) and mismatch repair deficiency (dMMR)/microsatellite instability (MSI), have been reported.^9^, ^10^, ^11^ Above all, PD‐L1 expression is considered a logical biomarker because PD‐L1 is a substantial target of the ICIs. An initial phase I study on the use of nivolumab, one of the PD‐1 inhibitors, supported a potential role for assessment of PD‐L1 expression on tumor cells in patients with several types of solid tumor including melanoma, NSCLC, renal cell carcinoma, and colorectal cancer (CRC).^12^ However, in contrast to the clinical use of PD‐L1 assessment in patients with melanoma and NSCLC,^13^, ^14^, ^15^ the clinical significance of PD‐L1 expression in GI cancer remains unclear from contradictory outcomes in multiple studies on the correlation between PD‐L1 expression and the ICI response or prognosis.

In this review, we focus on PD‐L1 expression in GI cancer and summarize its clinical significance as a prognostic biomarker and as a predictive biomarker for PD‐1 inhibitors. In addition, we discuss challenging issues for PD‐L1 assessment from the viewpoint of methodology for PD‐L1 evaluation, and biological features of PD‐L1 expression that display spatial and temporal heterogeneity, with future perspectives using liquid biopsy for better assessment of PD‐L1 status. This new information should improve our understanding of the clinical significance of PD‐L1 in GI cancer, leading to optimal patient selection and treatment strategy for the clinical use of PD‐1/PD‐L1 inhibitors in patients with GI cancer.

## 2 CLINICAL SIGNIFICANCE OF PD‐L1 EXPRESSION IN GI CANCER

### 2.1 Prognostic significance of PD‐L1 expression in GI cancer

Multiple studies on the relationship between PD‐L1 expression and patient survival in GI cancer have been reported. Recent studies involving large numbers of patients are summarized in Table 1.^16^, ^17^, ^18^, ^19^, ^20^, ^21^, ^22^, ^23^, ^24^, ^25^, ^26^, ^27^, ^28^, ^29^ Although the differences in patient background and assessment methods of PD‐L1 expression were major limitations, the prognostic significance of PD‐L1 expression in GI cancer was highly heterogeneous in each study. Most of the studies demonstrated that PD‐L1 positivity is a poor prognostic biomarker as PD‐L1 contributes to immune evasion. However, other recent studies did not confirm this; indeed, several studies indicated that PD‐L1 positivity is a better prognostic factor. The latter studies discussed that PD‐L1 expression does not necessarily represent an immunosuppressive state in the tumor microenvironment, but rather acts as a surrogate marker of immune activation because PD‐L1 is upregulated by some inflammatory cytokines such as interferon‐γ, which is secreted from activated immune effector cells.^30^ In fact, high PD‐L1 expression was associated with a high density of tumor‐infiltrating lymphocytes (TILs), which is regarded as the preferred state of the host immune response.^24^ In addition, some specific situations such as Epstein–Barr virus infection and dMMR in GI cancer are also reported to be associated with high PD‐L1 status.^31^, ^32^ Although these molecular subtypes and genetic profiles themselves have been reported to be promising predictive biomarkers for PD‐1 inhibitors,^33^, ^34^ prognostic prediction for such cases by assessment of PD‐L1 alone apparently remains difficult given the contradictory results from several studies.^35^, ^36^, ^37^

**TABLE 1:** Recent studies on the relationship between PD‐L1 expression and patient survival in GI cancer

Authors	Year	Journal	N	Ab Clone	Cut‐off value	PD‐L1 Positive rate	Prognostic outcome
*Esophageal cancer*							
Tanaka K et al^16^	2016	Cancer Sci	180	27A2	NA^a^	29.4%	Worse OS
Kim R et al^17^	2017	World J Gastroenterol	200	E1L3N	TCs > 10%	33.5%	No impact
Zhang W et al^18^	2017	Cancer Sci	344	SP142	TCs, ICs > 5%	TCs 14.5%, ICs 24.7%	Better OS and DFS (ICs)
Kollmann D et al^19^	2018	Oncoimmunology	168	E1L3N	TCs, TILs > 1%	TCs 43.5%, TILs 69%	Better OS and DFS (TCs, TILs)
Yagi T et al^20^	2019	Ann Surg	305	E1L3N	TCs > 25%^a^	17.4%	Worse OS and DFS
*Gastric cancer*							
Eto S et al^21^	2016	Gastric Cancer	105	EPR1161‐2	TCs > 50%	24.8%	Worse OS (NS)
Kim JW et al^22^	2016	Gastric Cancer	243	NA	TCs > 10%^a^	TCs 43.6%	Better OS and DFS
Dai C et al^23^	2016	Mol Oncol	444	MKP1A07310	TCs > 5%^a^	TCs 14.1%	Better OS (NS)
Kawazoe A et al^24^	2017	Gastric Cancer	487	SP142	TCs, ICs > 1%	TCs 12%, ICs 44%	No impact
Wang L et al^25^	2018	Cancer Med	550	28‐8	TCs, ICs > 1%	TCs 17.3%, ICs 34.5%	No impact
Yamashita K et al^26^	2020	Gastric Cancer	191	E1L3N	TPS, CPS > 1	TPS 20.4%, CPS 71.7%	Worse OS and DFS (CPS)
*Colorectal cancer*							
Lee LH et al^27^	2016	Mod Pathol	394	E1L3N	NA^a^	5%	No impact
Koganemaru S et al^28^	2017	Cancer Sci	235	SP142	TCs, ICs > 5%	TCs 8.1%, ICs 15.3%	Worse DFS (TCs), better DFS (ICs)
Huang CY et al^29^	2018	Sci Rep	867	28‐8	TCs > 5%	44%	Better DFS

Note

Abbreviations: CPS, Combined positive score; DFS, Disease‐free survival; ICs, Immune cells; NA, Not available; NS, Not statistically significant; OS, Overall survival; TCs, Tumor cells; TILs, tumor‐infiltrating lymphocytes; TPS, Tumor proportion score.

^a^ PD‐L1 positivity is defined by a combination of stained area and staining intensity in each study.

Taken together, the prognostic significance of PD‐L1 expression in GI cancer is still unknown. It may be challenging to consider PD‐L1 expression as the result of preferable host immune response or as a predisposition to cause immune evasion. Therefore, comprehensive analysis with other immune markers and immune profiles will be required to better assess the role of PD‐L1 expression as a prognostic factor.

### 2.2 Predictive role of PD‐L1 expression for PD‐1 inhibitors in GI cancer

To date, PD‐1/PD‐L1 inhibitors, such as nivolumab and pembrolizumab, for clinical use in GI cancer have been approved based on the results of several important clinical trials. The recent key trials on the efficacy of the PD‐1/PD‐L1 inhibitors in GI cancer are summarized in Table 2.^38^, ^39^, ^40^, ^41^, ^42^, ^43^, ^44^, ^45^, ^46^, ^47^, ^48^, ^49^ Here, we introduce the current clinical evidence and the significance of PD‐L1 expression as a predictive biomarker for the ICIs in each clinical trial. Several clinical trials on the efficacy of combination therapy of ICIs and PD‐L1 inhibitors are ongoing and are not discussed in detail here.

**TABLE 2:** Recent key trials on the efficacy of PD‐1/PD‐L1 inhibitors in GI cancer

Trial	Agent	N	Patients (treatment line)	Study design	Remarks
*Esophageal cancer (EC)*					
KEYNOTE‐028^38^ (P‐Ib)	Pem	23	ESCC, EAC, GEJ AC (≥2)	NA	ORR 30%, median OS 7.0 mo
KEYNOTE‐180^39^ (P‐II)	Pem	121	EC (≥3)	NA	ORR 13.8% (PD‐L1+), 6.3% (PD‐L1–)
KEYNOTE‐181^40^ (P‐III)	Pem	628	ESCC, EAC (≥2)	Pem vs. CT (PTX or DTX or IRI)	PD‐L1+, improved OS; ITT, no significance
ATTRACTION‐1^41^ (P‐II)	Niv	64	ESCC (≥2)	NA	ORR 17%, median OS 10.8 mo
ATTRACTION‐3^42^ (P‐III)	Niv	419	ESCC (≥2)	Niv vs. CT (PTX or DTX)	Niv improved OS regardless of PD‐L1 status
*Gastric and GEJ cancer (GC and GEJ C)*					
KEYNOTE‐012^43^ (P‐Ib)	Pem	39	GAC and GEJ C (NA)	NA	ORR 22%
KEYNOTE‐059^44^ (P‐II)	Pem	259	GC and GEJ C (≥2)	NA	ORR 15.5% (PD‐L1+), 6.4% (PD‐L1–)
KEYNOTE‐061^45^ (P‐III)	Pem	592	GC and GEJ C (≥2)	Pem vs. PTX	Pem did not significantly improve OS (≥CPS 1)
*Pem showed better OS (≥CPS 10)*					
ATTRACTION‐2^46^ (P‐III)	Niv	493	GC and GEJ C (≥3)	Niv vs. placebo	Niv improved OS regardless of PD‐L1 status
*(dMMR/MSI‐H) Colorectal cancer (CRC)*					
KEYNOTE‐016^47^ (P‐II)	Pem	41	dMMR or pMMR CRC (NA)	NA	ORR 40%; PD‐L1, not associated with PFS or OS
KEYNOTE‐164^48^ (P‐II)	Pem	128	dMMR/MSI‐H CRC (≥)	NA	ORR 33%
CheckMate 142^49^ (P‐II)	Niv	74	dMMR/MSI‐H CRC (≥2)	NA	ORR 29% (PD‐L1+), 28% (PD‐L1–)

Abbreviations: CPS, combined positive score; dMMR, mismatch repair deficient; DTX, docetaxel; EAC, esophageal adenocarcinoma; ESCC, esophageal squamous cell carcinoma; GAC, gastric adenocarcinoma; IRI, irinotecan; ITT, intention to treat; MSI‐H, microsatellite instability‐high; NA, not available; Niv, nivolumab; ORR, objective response rate; OS, overall survival; Pem, pembrolizumab; pMMR, mismatch repair procifient; PTX, paclitaxel.

#### 2.2.1 Esophageal cancer

First, the two single‐arm trials ATTRACTION‐01 and KEYNOTE‐028 were reported for esophageal cancer (EC). ATTRACTION‐01 was a phase II trial on the efficacy of nivolumab and showed 17% objective response rate (ORR) and 10.8 months for median overall survival (OS) for EC without PD‐L1 assessment.^41^ In contrast, patients with PD‐L1‐positive advanced solid tumors including EC were eligible in the phase Ib KEYNOTE‐028 trial, in which pembrolizumab showed 30% ORR and 7.0 months for median OS.^38^ Subsequently, phase II and III trials for the efficacy of nivolumab and pembrolizumab, respectively, were conducted. KEYNOTE‐180 was a phase II trial of the efficacy of pembrolizumab for patients with advanced or recurrent EC with two or more prior treatments. In this trial, PD‐L1 expression was evaluated by the combined positive score (CPS), which scores PD‐L1 expression on tumor cells and immune cells, with a cut‐off value of 10. The ORR was 13.8% among patients with PD‐L1+ tumors and 6.3% among patients with PD‐L1– tumors.^39^ Finally, the phase III KEYNOTE‐181 trial demonstrated that pembrolizumab significantly improved OS compared with chemotherapy in patients with advanced EC with PD‐L1 CPS ≥ 10 (median, 9.3 months vs 6.7 months; HR, 0.69; 95% CI, 0.52‐0.93; *P* = .0074), while median OS was 7.1 months for both treatment groups in the intention to treat group (HR, 0.89; 95% CI, 0.75‐1.05; *P* = .0560).^40^ These results support the predictive significance of PD‐L1 expression for pembrolizumab, and the US Food and Drug Administration (FDA) has approved pembrolizumab for patients with recurrent, locally advanced or metastatic EC with PD‐L1 of CPS ≥ 10 as 2 or more therapy line. On the other hand, the ATTRACTION‐3 trial, which was a phase III trial of the efficacy of nivolumab, demonstrated that nivolumab significantly improved OS compared with chemotherapy (median, 10.9 months for nivolumab vs 8.4 months for chemotherapy; HR, 0.77; 95% CI 0.62‐0.96; *P* = .019).^42^ However, in this trial, the survival benefit occurred regardless of PD‐L1 expression on the tumor with several cut‐off values of 1, 5, and 10, although patients with PD‐L1 ≥ 1%, tumor cells had a 15% greater reduction in the risk of death than those with PD‐L1 < 1%. Thus, nivolumab has just been approved in Japan for clinical use for all‐comer populations of advanced unresectable EC who received prior treatment.

#### 2.2.2 Gastric and GEJ cancer

For gastric cancer (GC) and gastroesophageal junction (GEJ) cancer, the initial phase Ib trial KEYNOTE‐012 showed 22% ORR in patient with PD‐L1 + advanced GC and triggered the initiation of further trials.^43^ In this trial, there was no association between the response to pembrolizumab and higher PD‐L1 expression on tumor cells, while a weak association between high PD‐L1 + mononuclear inflammatory cell densities and the response was observed. In the following phase II trial KEYNOTE‐059, the ORR of the pembrolizumab group was 11.6% in all enrolled patients, and durable responses were observed in patients with PD‐L1 + and PD‐L1– tumors with CPS ≥ 1 in subgroup analysis (PD‐L1 + ORR, 15.5% vs PD‐L1– ORR, 6.4%).^44^ Based on this trial, the FDA granted accelerated approval to pembrolizumab for patients with recurrent locally advanced or metastatic GC or GEJ adenocarcinoma with PD‐L1 CPS ≥ 1. Subsequently, the phase III KEYNOTE‐061 trial, which was a randomized controlled trial of pembrolizumab versus paclitaxel, was conducted.^45^ However, this trial failed to demonstrate a survival benefit for pembrolizumab compared with paclitaxel as second‐line therapy for patients with PD‐L1 CPS ≥ 1 (median OS, 9.1 months for pembrolizumab vs 8.4 months for paclitaxel; HR, 0.82; 95% CI 0.66‐1.03; one‐sided *P* = .0421). Notably, post‐hoc subgroup analyses suggested that the treatment effect of pembrolizumab was greater for patients with a PD‐L1 CPS ≥ 10 (median OS, 10.4 months for pembrolizumab vs 8.0 months for paclitaxel; HR, 0.64, 95% CI 0.41‐1.02). For nivolumab, the phase III ATTRACTION‐02 trial was conducted.^46^ This was a randomized, placebo‐controlled trial in an Asian population with GC or GEJ cancer who received two or more prior therapies. This trial demonstrated better OS in the nivolumab group than in the placebo group (median OS, 5.26 months for nivolumab vs 4.14 months for placebo; HR, 0.63, 95% CI 0.51‐0.78, *P* < .0001). However, the survival benefit was shown regardless of PD‐L1 positivity, defined as staining in 1% or more of tumor cells (PD‐L1+: median OS, 5.22 months for nivolumab vs 3.83 months for placebo, HR 0.51; PD‐L1–: median OS, 6.05 months for nivolumab vs 4.19 months for placebo, HR 0.72). Therefore, nivolumab was first approved in Japan for clinical use of ICIs for all‐comer populations of advanced GC and EGJ cancer who received at least two prior treatments. However, it should be noted that only about 40% of the samples were available for evaluation of PD‐L1 expression in this trial. Therefore, the predictive significance of PD‐L1 expression for nivolumab is unclear and further analysis is needed.

#### 2.2.3 Colorectal cancer (dMMR/MSI‐H colorectal cancer)

Although ICIs had been expected to be less effective for CRC, the KEYNOTE‐016 trial first demonstrated the effectiveness of pembrolizumab for patients with metastatic dMMR CRC.^47^ In this phase II trial, PD‐L1 expression was observed only in patients with dMMR cancer, and PD‐L1 expression was not significantly associated with patient survival. The subsequent phase II KEYNOTE‐164 trial demonstrated the efficacy of pembrolizumab for patients with MSI‐H/dMMR CRC (the ORR 33%).^48^ Unfortunately, this trial lacked any biomarker analysis, including PD‐L1 expression, due to limited tissue samples. Accordingly, the FDA first granted tissue/site‐agnostic approval to pembrolizumab for patients with unresectable or metastatic MSI‐H/dMMR solid tumors based on the results of the five trials (KEYNOTE‐016, KEYNOTE‐164, KEYNOTE‐012, KEYNOTE‐028, and KEYNOTE‐158^50^). However, the predictive value of PD‐L1 expression for pembrolizumab in MSI‐H/dMMR solid tumors remains unknown, and further analysis is needed. On the other hand, the efficacy of nivolumab in patients with metastatic MSI‐H/dMMR CRC was reported in the CheckMate 142 trial.^49^ In this trial, nivolumab monotherapy demonstrated durable response and disease control in pretreated patients with dMMR/MSI‐H metastatic CRC regardless of PD‐L1 expression (ORR, 29% for PD‐L1 ≥ 1% vs 28% for PD‐L1 < 1). Thus, the predictive significance of PD‐L1 expression for nivolumab, as well as for pembrolizumab, in dMMR/MSI‐H metastatic CRC is unestablished.

## 3 CLINICAL ISSUES FOR THE ASSESSMENT OF PD‐L1 EXPRESSION

For the assessment of PD‐L1 expression in clinical practice, immunohistochemistry staining (IHC) assays for PD‐L1 are usually performed using pretreatment tissue samples obtained by biopsy. However, several studies have pointed out that some clinical issues can affect the accurate assessment of PD‐L1 status in individuals. These issues are potentially associated with the significance of PD‐L1 expression as a prognostic or a predictive biomarker for PD‐1/PD‐L1 inhibitors. We discuss the clinical issues concerning PD‐L1 evaluation in terms of their technical and biological aspects.

### 3.1 Evaluation methods for PD‐L1 expression

#### 3.1.1 PD‐L1 IHC detecting assay

Various monoclonal primary antibodies against PD‐L1 with laboratory‐developed or standardized assays have been used in experimental studies and clinical trials (Table 3). Several studies noted that differences in primary antibodies and detection assay for PD‐L1 could affect the degree of tissue staining in NSCLC, melanoma, and GI cancer.^51^, ^52^, ^53^ Accordingly, standardized PD‐L1 IHC detecting assays for clinical use have been developed and approved by the FDA as a companion diagnostic for certain PD‐1/PD‐L1 inhibitors.^54^ In GI cancer, the PD‐L1 IHC 22C3 pharmDx assay was approved as a companion diagnostic for pembrolizumab in advanced EC (based on the KEYNOTE‐180 and KEYNOTE‐181 trials) and in advanced GC and GEJ cancer (based on the KEYNOTE‐059 trial). On the other hand, the 28‐8 pharmDx assay was used for PD‐L1 evaluation in clinical trials of nivolumab (ATTRACTION‐2, ATTRACTION‐3). Although some reports directly compared stainability between the two assays using the same tissue samples of NSCLC,^55^ few studies made such a comparison using the same samples of GI cancer. Further comparative studies will be required for accurate diagnosis of PD‐L1 expression.

**TABLE 3:** PD‐L1 IHC assay in recent key trials of the efficacy of PD‐1/PD‐L1 inhibitors in GI cancer

Trial	Agent	N	PD‐L1 IHC assay	Cut‐off value	PD‐L1 positivity
*Esophageal cancer*					
KEYNOTE‐028^38^ (P‐Ib)	Pem	23	22C3 laboratory‐developed testing	> 1% scorable cells	All patients
KEYNOTE‐180^39^ (P‐II)	Pem	121	22C3 pharmDx assay	> CPS 10	47.9%
KEYNOTE‐181^40^ (P‐III)	Pem	628	22C3 pharmDx assay	> CPS 10	35.4%
ATTRACTION‐1^41^ (P‐II)	Niv	64	NA	NA	NA
ATTRACTION‐3^42^ (P‐III)	Niv	419	28‐8 pharmDx assay	> 1% TCs	48.4%
*Gastric and GEJ cancer*					
KEYNOTE‐012^43^ (P‐Ib)	Pem	39	22C3 pharmDx assay	> 1% scorable cells	All patients
KEYNOTE‐059^44^ (P‐II)	Pem	259	22C3 pharmDx assay	> CPS 1	57.1%
KEYNOTE‐061^45^ (P‐III)	Pem	592	22C3 pharmDx assay	> CPS 1	66.7%
ATTRACTION‐2^46^ (P‐III)	Niv	493	28‐8 pharmDx assay	> 1% TCs	13.5%
*(dMMR/MSI‐H) Colorectal cancer*					
KEYNOTE‐016^47^ (P‐II)	Pem	41	NA	NA	NA
KEYNOTE‐164^48^ (P‐II)	Pem	128	NA	NA	NA
CheckMate 142^49^ (P‐II)	Niv	74	28‐8 pharmDx assay	> 1% TCs	30.9%

Abbreviations: CPS, combined positive score; dMMR, mismatch repair deficient; NA, not available; Niv, nivolumab; Pem, pembrolizumab; pMMR, mismatch repair procifient; TCs, tumor cells.

#### 3.1.2 Assessment methods for PD‐L1 expression

Classically, the assessment methods for PD‐L1 expression varied in each study. Some studies used their own criteria based on a combination of stained area and staining intensity, while others evaluated the percentage of stained cells with several cut‐off values. These distinct criteria should be associated with a wide range of PD‐L1 positivity even in the same cancer type. Thus, two scoring systems have been adopted in clinical trials: the percentage of stained tumor cells, which is substantially the same as the tumor proportion score (TPS) used in NSCLC, and CPS, which assesses all PD‐L1+ cells including tumor cells, lymphocytes, and macrophages (Table 3). Although it is not established which of these is better for the assessment of PD‐L1 expression, several studies have indicated the usefulness of CPS.^56^ We recently demonstrated the utility of CPS as a prognostic biomarker in GC.^26^ Interestingly, Herbst et al reported that the PD‐1/PD‐L1 pathway was inhibited, especially when PD‐L1 was expressed by tumor‐infiltrating immune cells.^57^ From these considerations and the results of recent clinical trials, CPS may become the standard method for PD‐L1 evaluation as a prognostic and predictive biomarker.

Elucidation of the optimal cut‐off value is another concern. A meta‐analysis in solid tumors demonstrated a positive dose‐response relationship between PD‐L1 positivity (with cut‐off value of 1, 5, and 10) and survival benefit.^58^ As with NSCLC, in which TPS with cut‐off value of 50% is adopted for clinical use of PD‐1 inhibitors, future studies will determine optimal organ‐specific cut‐off values of PD‐L1 expression in GI cancer.

### 3.2 Tumor heterogeneity of PD‐L1 expression

Tumor heterogeneity is one of the most crucial hallmarks of cancer.^59^ As a result of this heterogeneity, the bulk tumor may attain a diversity of distinct molecular features with differential levels of sensitivity to treatment.^60^ Moreover, tumor heterogeneity often poses substantial issues in biomarker analysis, and thus a better understanding of this issue should have important implications for clinical practice.

#### 3.2.1 Spatial heterogeneity of PD‐L1 expression

**FIGURE 1:**
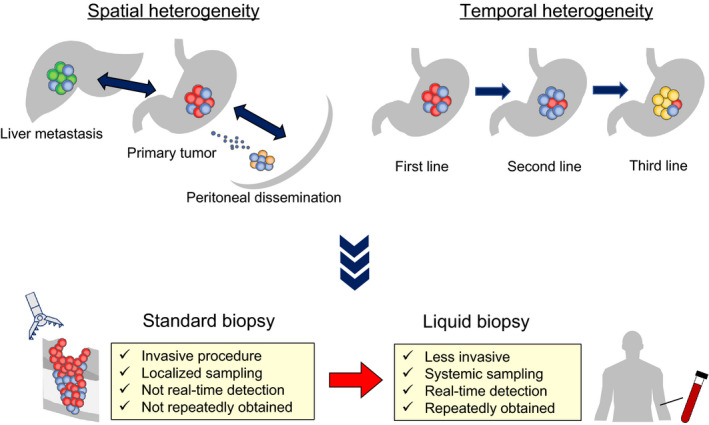
Spatial and temporal heterogeneity of PD‐L1 expression. Liquid biopsy is a promising option to solve several clinical issues associated with tumor heterogeneity

Spatial heterogeneity is described as the non‐uniform distribution of diverse tumor subpopulations within a single disease site or across different disease sites (Figure 1). For PD‐L1 expression, several publications have demonstrated spatial heterogeneity within a primary site in GI cancer.^61^, ^62^ This spatial heterogeneity raises the important clinical question of whether a standard biopsy from the primary tumor site can reflect the PD‐L1 expression of the whole tumor bulk in a patient. Indeed, we recently reported that the low concordance rate of PD‐L1 expression between biopsy and resected samples from the same GC cases and single biopsy was associated with such a discordance.^63^ Moreover, Van den Eynde et al also demonstrated the heterogeneous immune diversity between primary and metastatic CRC and the inaccuracy of PD‐L1 evaluation by single biopsy.^64^ Importantly, the spatial heterogeneity of PD‐L1 expression between primary and metastatic tumor is also reported in NSCLC and breast cancer,^65^, ^66^ in which evaluation of PD‐L1 expression is required for clinical use of PD‐1 inhibitors. Therefore, such spatial heterogeneity should be considered in the treatments with PD‐1 inhibitors for GI cancer as well. Although multiple biopsies may cover the heterogeneity, the optimal number of biopsies and the need for biopsies for metastatic lesions are pivotal issues to be considered.

#### 3.2.2 Temporal heterogeneity of PD‐L1 expression

Temporal heterogeneity is described as the dynamic variation in the genetic diversity during the course of disease. In addition to genetic and phenotypic alterations in the process of tumor progression, the effect of cancer therapies on PD‐L1 expression should be considered (Figure 1). Several reports have demonstrated that some cytotoxic agents including fluorouracil, paclitaxel, and radiation therapy can upregulate PD‐L1 expression via cell signaling pathways in GI cancer.^67^, ^68^, ^69^ Notably, Yang et al reported that GC patients with a preferable response to chemotherapy displayed PD‐L1 downregulation and showed better RFS, whereas pretreatment PD‐L1 status was not associated with survival.^70^ Ogura et al also demonstrated equivalent results in patients with rectal cancer who received CRT.^71^ These impressive results suggest the importance of temporal assessment of PD‐L1 expression during the course of treatment. Nevertheless, previous clinical trials on the efficacy of PD‐1/PD‐L1 inhibitors for patients who received prior therapies used pretreatment samples for PD‐L1 assessment. Therefore, a re‐biopsy strategy should be established to account for the current PD‐L1 status after treatment, leading to a better appreciation of the true significance of PD‐L1 expression as a biomarker.

## 4 FUTURE PERSPECTIVES

To overcome the clinical issues described above for the assessment of PD‐L1 expression, several strategies have been considered, including the development of companion diagnostics, as well as multiple and repeated biopsies from both primary and metastatic tumors. However, this may not be feasible in clinical practice, as tissue biopsies are limited to very few sampling points and accessible metastatic sites. Given this situation, liquid biopsy (LB) is a promising option to solve these problems because LB is less invasive, more systemic, and can be obtained at multiple time points during the treatment course (Figure 1).^72^ LB includes a variety of analytes such as circulating tumor DNA, cell‐free RNA, and circulating tumor cells (CTCs). Among them, PD‐L1 expression on CTCs can be a potential predictive biomarker for PD‐1 inhibitors. In fact, several studies have demonstrated that PD‐L1 status in CTCs correlated with PD‐L1 status in tumor tissue and helps to predict the therapeutic effect of PD‐1 inhibitors in NSCLC and melanoma.^73^, ^74^ For GI cancer, Yue et al reported that the abundance of PD‐L1^high^ CTCs was a predictive biomarker of PD‐1/PD‐L1 inhibitors and the dynamic changes of PD‐L1^high^ CTCs correlated with disease outcomes.^75^ In addition, assessment of systemic PD‐L1 status using other analytes as LB may be applicable. Chen et al reported the utility of exosomal PD‐L1 on extracellular vesicules as a predictive biomarker for PD‐1 inhibitors in melanoma.^76^ Moreover, soluble PD‐L1 is also reportedly an available analyte for survival analysis in GI cancer.^77^, ^78^, ^79^ Interestingly, a functional analysis described by Takeuch et al demonstrated glycosylation of soluble PD‐L1 plays an important role in its binding to PD‐1 receptor.^80^ Such functional analysis may be help for biomarker analysis of PD‐L1 using LB. Although LB has some limitations such as low detection rate and unestablished standard protocols for clinical use,^81^ further studies will reveal clinical utilities of LB in assessment of PD‐L1 expression in GI cancer.

## 5 CONCLUSIONS

We reviewed the current evidence for PD‐L1 expression as a prognostic and predictive biomarker for PD‐1/PD‐L1 inhibitors. Although PD‐L1 is a promising biomarker, some associated clinical issues remain to be addressed. Accurate assessment of PD‐L1 expression will reveal its true clinical significance and lead to the establishment of more effective strategies for the clinical use of ICIs.

## DISCLOSURE

Conflict of Interest: The authors declare no conflict of interest.

Funding: This work was supported in part by the following grant and foundation: Japan Society for the Promotion of Science, Grant‐in‐Aid for Scientific Research; Grant no. 20K16308.

## References

[1] Chen DS , Mellman I . Oncology meets immunology: the cancer‐immunity cycle. Immunity. 2013;39:1–10. 23890059 10.1016/j.immuni.2013.07.012

[2] Sanmamed MF , Chen L . A paradigm shift in cancer immunotherapy: from enhancement to normalization. Cell. 2018;175:313–26. 30290139 10.1016/j.cell.2018.09.035 PMC6538253

[3] Pardoll DM . The blockade of immune checkpoints in cancer immunotherapy. Nat Rev Cancer. 2012;12:252–64. 22437870 10.1038/nrc3239 PMC4856023

[4] Ishida Y , Agata Y , Shibahara K , Honjo T . Induced expression of PD‐1, a novel member of the immunoglobulin gene superfamily, upon programmed cell death. EMBO J. 1992;11:3887–95. 1396582 10.1002/j.1460-2075.1992.tb05481.x PMC556898

[5] Brahmer JR , Tykodi SS , Chow LQM , Hwu W‐J , Topalian SL , Hwu P , et al. Safety and activity of anti‐PD‐L1 antibody in patients with advanced cancer. N Engl J Med. 2012;366:2455–65. 22658128 10.1056/NEJMoa1200694 PMC3563263

[6] Wolchok JD , Kluger H , Callahan MK , Postow MA , Rizvi NA , Lesokhin AM , et al. Nivolumab plus ipilimumab in advanced melanoma. N Engl J Med. 2013;369:122–33. 23724867 10.1056/NEJMoa1302369 PMC5698004

[7] Vokes EE , Ready N , Felip E , Horn L , Burgio MA , Antonia SJ , et al. Nivolumab versus docetaxel in previously treated advanced non‐small‐cell lung cancer (CheckMate 017 and CheckMate 057): 3‐year update and outcomes in patients with liver metastases. Ann Oncol. 2018;29:959–65. 29408986 10.1093/annonc/mdy041

[8] Iwai Y , Ishida M , Tanaka Y , Okazaki T , Honjo T , Minato N . Involvement of PD‐L1 on tumor cells in the escape from host immune system and tumor immunotherapy by PD‐L1 blockade. Proc Natl Acad Sci U S A. 2002;99:12293–7. 12218188 10.1073/pnas.192461099 PMC129438

[9] Gibney GT , Weiner LM , Atkins MB . Predictive biomarkers for checkpoint inhibitor‐based immunotherapy. Lancet Oncol. 2016;17:e542–e551. 27924752 10.1016/S1470-2045(16)30406-5 PMC5702534

[10] Topalian SL , Taube JM , Anders RA , Pardoll DM . Mechanism‐driven biomarkers to guide immune checkpoint blockade in cancer therapy. Nat Rev Cancer. 2016;16:275–87. 27079802 10.1038/nrc.2016.36 PMC5381938

[11] Havel JJ , Chowell D , Chan TA . The evolving landscape of biomarkers for checkpoint inhibitor immunotherapy. Nat Rev Cancer. 2019;19:133–50. 30755690 10.1038/s41568-019-0116-x PMC6705396

[12] Topalian SL , Hodi FS , Brahmer JR , Gettinger SN , Smith DC , McDermott DF , et al. Safety, activity, and immune correlates of anti‐PD‐1 antibody in cancer. N Engl J Med. 2012;366:2443–54. 22658127 10.1056/NEJMoa1200690 PMC3544539

[13] Larkin J , Chiarion‐Sileni V , Gonzalez R , Grob JJ , Cowey CL , Lao CD , et al. Combined nivolumab and ipilimumab or monotherapy in untreated melanoma. N Engl J Med. 2015;373:23–34. 26027431 10.1056/NEJMoa1504030 PMC5698905

[14] Garon EB , Rizvi NA , Hui R , Leighl N , Balmanoukian AS , Eder JP , et al. Pembrolizumab for the treatment of non‐small‐cell lung cancer. N Engl J Med. 2015;372:2018–28. 25891174 10.1056/NEJMoa1501824

[15] Borghaei H , Paz‐Ares L , Horn L , Spigel DR , Steins M , Ready NE , et al. Nivolumab versus docetaxel in advanced nonsquamous non‐small‐cell lung cancer. N Engl J Med. 2015;373:1627–39. 26412456 10.1056/NEJMoa1507643 PMC5705936

[16] Tanaka K , Miyata H , Sugimura K , Kanemura T , Hamada‐Uematsu M , Mizote YU , et al. Negative influence of programmed death‐1‐ligands on the survival of esophageal cancer patients treated with chemotherapy. Cancer Sci. 2016;107:726–33. 27015293 10.1111/cas.12938 PMC4968603

[17] Kim R , Keam B , Kwon D , Ock C‐Y , Kim M , Kim TM , et al. Programmed death ligand‐1 expression and its prognostic role in esophageal squamous cell carcinoma. World J Gastroenterol. 2016;22:8389–97. 27729745 10.3748/wjg.v22.i37.8389 PMC5055869

[18] Zhang W , Pang Q , Zhang X , Yan C , Wang Q , Yang J , et al. Programmed death‐ligand 1 is prognostic factor in esophageal squamous cell carcinoma and is associated with epidermal growth factor receptor. Cancer Sci. 2017;108:590–7. 28192623 10.1111/cas.13197 PMC5406530

[19] Kollmann D , Ignatova D , Jedamzik J , Chang Y‐T , Jomrich G , Baierl A , et al. PD‐L1 expression is an independent predictor of favorable outcome in patients with localized esophageal adenocarcinoma. Oncoimmunology. 2018;7:e1435226. 29872575 10.1080/2162402X.2018.1435226 PMC5980377

[20] Yagi T , Baba Y , Ishimoto T , Iwatsuki M , Miyamoto Y , Yoshida N , et al. PD‐L1 expression, tumor‐infiltrating lymphocytes, and clinical outcome in patients with surgically resected esophageal cancer. Ann Surg. 2019;269:471–8. 29206673 10.1097/SLA.0000000000002616

[21] Eto S , Yoshikawa K , Nishi M , Higashijima J , Tokunaga T , Nakao T , et al. Programmed cell death protein 1 expression is an independent prognostic factor in gastric cancer after curative resection. Gastric Cancer. 2016;19:466–71. 26210691 10.1007/s10120-015-0519-7

[22] Kim JW , Nam KH , Ahn S‐H , Park DJ , Kim H‐H , Kim SH , et al. Prognostic implications of immunosuppressive protein expression in tumors as well as immune cell infiltration within the tumor microenvironment in gastric cancer. Gastric Cancer. 2016;19:42–52. 25424150 10.1007/s10120-014-0440-5

[23] Dai C , Geng R , Wang C , Wong A , Qing M , Hu J , et al. Concordance of immune checkpoints within tumor immune contexture and their prognostic significance in gastric cancer. Mol Oncol. 2016;10:1551–8. 27720576 10.1016/j.molonc.2016.09.004 PMC5423138

[24] Kawazoe A , Kuwata T , Kuboki Y , Shitara K , Nagatsuma AK , Aizawa M , et al. Clinicopathological features of programmed death ligand 1 expression with tumor‐infiltrating lymphocyte, mismatch repair, and Epstein‐Barr virus status in a large cohort of gastric cancer patients. Gastric Cancer. 2017;20:407–15. 27629881 10.1007/s10120-016-0631-3

[25] Wang L , Zhang Q , Ni S , Tan C , Cai XU , Huang D , et al. Programmed death‐ligand 1 expression in gastric cancer: correlation with mismatch repair deficiency and HER2‐negative status. Cancer Med. 2018;7:2612–20. 29673110 10.1002/cam4.1502 PMC6010739

[26] Yamashita K , Iwatsuki M , Harada K , Eto K , Hiyoshi Y , Ishimoto T , et al. Prognostic impacts of the combined positive score and the tumor proportion score for programmed death ligand‐1 expression by double immunohistochemical staining in patients with advanced gastric cancer. Gastric Cancer. 2020;23:95–104. 31451991 10.1007/s10120-019-00999-9

[27] Lee LH , Cavalcanti MS , Segal NH , Hechtman JF , Weiser MR , Smith JJ , et al. Patterns and prognostic relevance of PD‐1 and PD‐L1 expression in colorectal carcinoma. Mod Pathol. 2016;29:1433–42. 27443512 10.1038/modpathol.2016.139 PMC5083129

[28] Koganemaru S , Inoshita N , Miura Y , Miyama YU , Fukui Y , Ozaki Y , et al. Prognostic value of programmed death‐ligand 1 expression in patients with stage III colorectal cancer. Cancer Sci. 2017;108:853–8. 28267224 10.1111/cas.13229 PMC5448596

[29] Huang C‐Y , Chiang S‐F , Ke T‐W , Chen T‐W , You Y‐S , Chen W‐L , et al. Clinical significance of programmed death 1 ligand‐1 (CD274/PD‐L1) and intra‐tumoral CD8+ T‐cell infiltration in stage II‐III colorectal cancer. Sci Rep. 2018;8:15658. 30353144 10.1038/s41598-018-33927-5 PMC6199287

[30] Chen J , Jiang CC , Jin L , Zhang XD . Regulation of PD‐L1: a novel role of pro‐survival signalling in cancer. Ann Oncol. 2016;27:409–16. 26681673 10.1093/annonc/mdv615

[31] Cancer Genome Atlas Research Network . Comprehensive molecular characterization of gastric adenocarcinoma. Nature. 2014;513:202–9. 25079317 10.1038/nature13480 PMC4170219

[32] Llosa NJ , Cruise M , Tam A , Wicks EC , Hechenbleikner EM , Taube JM , et al. The vigorous immune microenvironment of microsatellite instable colon cancer is balanced by multiple counter‐inhibitory checkpoints. Cancer Discov. 2015;5:43–51. 25358689 10.1158/2159-8290.CD-14-0863 PMC4293246

[33] Kim ST , Cristescu R , Bass AJ , Kim K‐M , Odegaard JI , Kim K , et al. Comprehensive molecular characterization of clinical responses to PD‐1 inhibition in metastatic gastric cancer. Nat Med. 2018;24:1449–58. 30013197 10.1038/s41591-018-0101-z

[34] Dudley JC , Lin MT , Le DT , Eshleman JR . Microsatellite instability as a biomarker for PD‐1 blockade. Clin Cancer Res. 2016;22:813–20. 26880610 10.1158/1078-0432.CCR-15-1678

[35] Seo AN , Kang BW , Kwon OK , Park KB , Lee SS , Chung HY , et al. Intratumoural PD‐L1 expression is associated with worse survival of patients with Epstein‐Barr virus‐associated gastric cancer. Br J Cancer. 2017;117:1753–60. 29073638 10.1038/bjc.2017.369 PMC5729479

[36] Sundar R , Qamra A , Tan ALK , Zhang S , Ng CCY , Teh BT , et al. Transcriptional analysis of immune genes in Epstein‐Barr virus‐associated gastric cancer and association with clinical outcomes. Gastric Cancer. 2018;21:1064–70. 29915957 10.1007/s10120-018-0851-9

[37] Saito R , Abe H , Kunita A , Yamashita H , Seto Y , Fukayama M . Overexpression and gene amplification of PD‐L1 in cancer cells and PD‐L1(+) immune cells in Epstein‐Barr virus‐associated gastric cancer: the prognostic implications. Mod Pathol. 2017;30:427–39. 27934877 10.1038/modpathol.2016.202

[38] Doi T , Piha‐Paul SA , Jalal SI , Saraf S , Lunceford J , Koshiji M , et al. Safety and antitumor activity of the anti‐programmed death‐1 antibody pembrolizumab in patients with advanced esophageal carcinoma. J Clin Oncol. 2018;36:61‐7. 29116900 10.1200/JCO.2017.74.9846

[39] Shah MA , Kojima T , Hochhauser D , Enzinger P , Raimbourg J , Hollebecque A , et al. Efficacy and safety of pembrolizumab for heavily pretreated patients with advanced, metastatic adenocarcinoma or squamous cell carcinoma of the esophagus: the Phase 2 KEYNOTE‐180 Study. JAMA Oncol. 2019;5:546–50. 30570649 10.1001/jamaoncol.2018.5441 PMC6459121

[40] Metges J , Francois E , Shah M , Adenis A , Enzinger P , Kojima T , et al. The phase 3 KEYNOTE‐181 study: pembrolizumab versus chemotherapy as second‐line therapy for advanced esophageal cancer. Ann Oncol. 2019;30(Suppl 4):iv130.

[41] Kudo T , Hamamoto Y , Kato K , Ura T , Kojima T , Tsushima T , et al. Nivolumab treatment for oesophageal squamous‐cell carcinoma: an open‐label, multicentre, phase 2 trial. Lancet Oncol. 2017;18:631–9. 28314688 10.1016/S1470-2045(17)30181-X

[42] Kato K , Cho BC , Takahashi M , Okada M , Lin C‐Y , Chin K , et al. Nivolumab versus chemotherapy in patients with advanced oesophageal squamous cell carcinoma refractory or intolerant to previous chemotherapy (ATTRACTION‐3): a multicentre, randomised, open‐label, phase 3 trial. Lancet Oncol. 2019;20:1506–17. 31582355 10.1016/S1470-2045(19)30626-6

[43] Muro K , Chung HC , Shankaran V , Geva R , Catenacci D , Gupta S , et al. Pembrolizumab for patients with PD‐L1‐positive advanced gastric cancer (KEYNOTE‐012): a multicentre, open‐label, phase 1b trial. Lancet Oncol. 2016;17:717–26. 27157491 10.1016/S1470-2045(16)00175-3

[44] Fuchs CS , Doi T , Jang RW , Muro K , Satoh T , Machado M , et al. Safety and efficacy of pembrolizumab monotherapy in patients with previously treated advanced gastric and gastroesophageal junction cancer: Phase 2 clinical KEYNOTE‐059 trial. JAMA Oncol. 2018;4:e180013. 29543932 10.1001/jamaoncol.2018.0013 PMC5885175

[45] Shitara K , Özgüroğlu M , Bang Y‐J , Di Bartolomeo M , Mandalà M , Ryu M‐H , et al. Pembrolizumab versus paclitaxel for previously treated, advanced gastric or gastro‐oesophageal junction cancer (KEYNOTE‐061): a randomised, open‐label, controlled, phase 3 trial. Lancet. 2018;392:123–33. 29880231 10.1016/S0140-6736(18)31257-1

[46] Kang Y‐K , Boku N , Satoh T , Ryu M‐H , Chao Y , Kato K , et al. Nivolumab in patients with advanced gastric or gastro‐oesophageal junction cancer refractory to, or intolerant of, at least two previous chemotherapy regimens (ONO‐4538‐12, ATTRACTION‐2): a randomised, double‐blind, placebo‐controlled, phase 3 trial. Lancet. 2017;390:2461–71. 28993052 10.1016/S0140-6736(17)31827-5

[47] Le DT , Uram JN , Wang H , Bartlett BR , Kemberling H , Eyring AD , et al. PD‐1 blockade in tumors with mismatch‐repair deficiency. N Engl J Med. 2015;372:2509–20. 26028255 10.1056/NEJMoa1500596 PMC4481136

[48] Le DT , Kim TW , Van Cutsem E , Geva R , Jäger D , Hara H , et al. Phase II open‐label study of pembrolizumab in treatment‐refractory, microsatellite instability‐high/mismatch repair‐deficient metastatic colorectal cancer: KEYNOTE‐164. J Clin Oncol. 2020;38:11–9. 31725351 10.1200/JCO.19.02107 PMC7031958

[49] Overman MJ , McDermott R , Leach JL , Lonardi S , Lenz H‐J , Morse MA , et al. Nivolumab in patients with metastatic DNA mismatch repair‐deficient or microsatellite instability‐high colorectal cancer (CheckMate 142): an open‐label, multicentre, phase 2 study. Lancet Oncol. 2017;18:1182–91. 28734759 10.1016/S1470-2045(17)30422-9 PMC6207072

[50] Marabelle A , Le DT , Ascierto PA , Di Giacomo AM , De Jesus‐Acosta A , Delord J‐P , et al. Efficacy of pembrolizumab in patients with noncolorectal high microsatellite instability/mismatch repair‐deficient cancer: results from the Phase II KEYNOTE‐158 Study. J Clin Oncol. 2020;38:1–10. 31682550 10.1200/JCO.19.02105 PMC8184060

[51] Adam J , Le Stang N , Rouquette I , Cazes A , Badoual C , Pinot‐Roussel H , et al. Multicenter harmonization study for PD‐L1 IHC testing in non‐small‐cell lung cancer. Ann Oncol. 2018;29:953–8. 29351573 10.1093/annonc/mdy014

[52] Sunshine JC , Nguyen PL , Kaunitz GJ , Cottrell TR , Berry S , Esandrio J , et al. PD‐L1 expression in melanoma: a quantitative immunohistochemical antibody comparison. Clin Cancer Res. 2017;23:4938–44. 28428193 10.1158/1078-0432.CCR-16-1821 PMC6175606

[53] Ma J , Li J , Qian M , Han W , Tian M , Li Z , et al. PD‐L1 expression and the prognostic significance in gastric cancer: a retrospective comparison of three PD‐L1 antibody clones (SP142, 28–8 and E1L3N). Diagn Pathol. 2018;13:91. 30463584 10.1186/s13000-018-0766-0 PMC6249875

[54] Davis AA , Patel VG . The role of PD‐L1 expression as a predictive biomarker: an analysis of all US Food and Drug Administration (FDA) approvals of immune checkpoint inhibitors. J Immunother Cancer. 2019;7:278. 31655605 10.1186/s40425-019-0768-9 PMC6815032

[55] Saito T , Tsuta K , Ishida M , Ryota H , Takeyasu Y , Fukumoto KJ , et al. Comparative study of programmed cell death ligand‐1 immunohistochemistry assays using 22C3 and 28–8 antibodies for non‐small cell lung cancer: analysis of 420 surgical specimens from Japanese patients. Lung Cancer. 2018;125:230–7. 30429026 10.1016/j.lungcan.2018.10.005

[56] Kulangara K , Zhang N , Corigliano E , Guerrero L , Waldroup S , Jaiswal D , et al. Clinical utility of the combined positive score for programmed death ligand‐1 expression and the approval of pembrolizumab for treatment of gastric cancer. Arch Pathol Lab Med. 2019;143:330–7. 30028179 10.5858/arpa.2018-0043-OA

[57] Herbst RS , Soria J‐C , Kowanetz M , Fine GD , Hamid O , Gordon MS , et al. Predictive correlates of response to the anti‐PD‐L1 antibody MPDL3280A in cancer patients. Nature. 2014;515:563–7. 25428504 10.1038/nature14011 PMC4836193

[58] Liu XI , Guo C‐Y , Tou F‐F , Wen X‐M , Kuang Y‐K , Zhu Q , et al. Association of PD‐L1 expression status with the efficacy of PD‐1/PD‐L1 inhibitors and overall survival in solid tumours: a systematic review and meta‐analysis. Int J Cancer. 2020;147(1):116–27. 31633798 10.1002/ijc.32744

[59] Hanahan D , Weinberg RA . Hallmarks of cancer: the next generation. Cell. 2011;144:646–74. 21376230 10.1016/j.cell.2011.02.013

[60] Dagogo‐Jack I , Shaw AT . Tumour heterogeneity and resistance to cancer therapies. Nat Rev Clin Oncol. 2018;15:81–94. 29115304 10.1038/nrclinonc.2017.166

[61] Yan T , Cui H , Zhou Y , Yang B , Kong P , Zhang Y , et al. Multi‐region sequencing unveils novel actionable targets and spatial heterogeneity in esophageal squamous cell carcinoma. Nat Commun. 2019;10:1670. 30975989 10.1038/s41467-019-09255-1 PMC6459928

[62] Hatogai K , Fujii S , Kitano S , Kojima T , Daiko H , Yoshino T , et al. Relationship between the immune microenvironment of different locations in a primary tumour and clinical outcomes of oesophageal squamous cell carcinoma. Br J Cancer. 2020;122:413–20. 31761900 10.1038/s41416-019-0622-3 PMC7000821

[63] Yamashita K , Iwatsuki M , Harada K , Koga Y , Kiyozumi Y , Eto K , et al. Can PD‐L1 expression evaluated by biopsy sample accurately reflect its expression in the whole tumour in gastric cancer? Br J Cancer. 2019;121:278–80. 31285589 10.1038/s41416-019-0515-5 PMC6738080

[64] Van den Eynde M , Mlecnik B , Bindea G , Fredriksen T , Church SE , Lafontaine L , et al. The link between the multiverse of immune microenvironments in metastases and the survival of colorectal cancer patients. Cancer Cell. 2018;34: 1012–26.e3. 30537506 10.1016/j.ccell.2018.11.003

[65] Cimino‐Mathews A , Thompson E , Taube JM , Ye X , Lu Y , Meeker A , et al. PD‐L1 (B7–H1) expression and the immune tumor microenvironment in primary and metastatic breast carcinomas. Hum Pathol. 2016;47:52–63. 26527522 10.1016/j.humpath.2015.09.003 PMC4778421

[66] Takamori S , Takada K , Tagawa T , Toyokawa G , Hirai F , Yamashita N , et al. Differences in PD‐L1 expression on tumor and immune cells between lung metastases and corresponding primary tumors. Surg Oncol. 2018;27:637–41. 30449485 10.1016/j.suronc.2018.08.001

[67] Van Der Kraak L , Goel G , Ramanan K , Kaltenmeier C , Zhang L , Normolle DP , et al. 5‐Fluorouracil upregulates cell surface B7–H1 (PD‐L1) expression in gastrointestinal cancers. J Immunother Cancer. 2016;4:65. 27777774 10.1186/s40425-016-0163-8 PMC5067917

[68] Zerdes I , Matikas A , Bergh J , Rassidakis GZ , Foukakis T . Genetic, transcriptional and post‐translational regulation of the programmed death protein ligand 1 in cancer: biology and clinical correlations. Oncogene. 2018;37:4639–61. 29765155 10.1038/s41388-018-0303-3 PMC6107481

[69] Kelly RJ , Zaidi AH , Smith MA , Omstead AN , Kosovec JE , Matsui D , et al. The dynamic and transient immune microenvironment in locally advanced esophageal adenocarcinoma post chemoradiation. Ann Surg. 2018;268:992–9. 28806299 10.1097/SLA.0000000000002410

[70] Yang JH , Kim H , Roh SY , Lee MA , Park JM , Lee HH , et al. Discordancy and changes in the pattern of programmed death ligand 1 expression before and after platinum‐based chemotherapy in metastatic gastric cancer. Gastric Cancer. 2019;22:147–54. 29860599 10.1007/s10120-018-0842-x

[71] Ogura A , Akiyoshi T , Yamamoto N , Kawachi H , Ishikawa Y , Mori S , et al. Pattern of programmed cell death‐ligand 1 expression and CD8‐positive T‐cell infiltration before and after chemoradiotherapy in rectal cancer. Eur J Cancer. 2018;91:11–20. 29328976 10.1016/j.ejca.2017.12.005

[72] Keller L , Pantel K . Unravelling tumour heterogeneity by single‐cell profiling of circulating tumour cells. Nat Rev Cancer. 2019;19:553–67. 31455893 10.1038/s41568-019-0180-2

[73] Ilié M , Szafer‐Glusman E , Hofman V , Chamorey E , Lalvée S , Selva E , et al. Detection of PD‐L1 in circulating tumor cells and white blood cells from patients with advanced non‐small‐cell lung cancer. Ann Oncol. 2018;29:193–9. 29361135 10.1093/annonc/mdx636

[74] Khattak MA , Reid A , Freeman J , Pereira M , McEvoy A , Lo J , et al. PD‐L1 expression on circulating tumor cells may be predictive of response to pembrolizumab in advanced melanoma: results from a pilot study. Oncologist. 2020;25:e520–e527. 32162809 10.1634/theoncologist.2019-0557 PMC7066715

[75] Yue C , Jiang Y , Li P , Wang Y , Xue J , Li N , et al. Dynamic change of PD‐L1 expression on circulating tumor cells in advanced solid tumor patients undergoing PD‐1 blockade therapy. Oncoimmunology. 2018;7:e1438111. 29900038 10.1080/2162402X.2018.1438111 PMC5993493

[76] Chen G , Huang AC , Zhang W , Zhang G , Wu M , Xu W , et al. Exosomal PD‐L1 contributes to immunosuppression and is associated with anti‐PD‐1 response. Nature. 2018;560:382–6. 30089911 10.1038/s41586-018-0392-8 PMC6095740

[77] Ito M , Oshima Y , Yajima S , Suzuki T , Nanami T , Shiratori F , et al. Is high serum programmed death ligand 1 level a risk factor for poor survival in patients with gastric cancer? Ann Gastroenterol Surg. 2018;2:313–8. 30003194 10.1002/ags3.12175 PMC6036390

[78] Shigemori T , Toiyama Y , Okugawa Y , Yamamoto A , Yin C , Narumi A , et al. Soluble PD‐L1 expression in circulation as a predictive marker for recurrence and prognosis in gastric cancer: Direct comparison of the clinical burden between tissue and serum PD‐L1 expression. Ann Surg Oncol. 2019;26:876–83. 10.1245/s10434-018-07112-x 30565045

[79] Ito M , Yajima S , Suzuki T , Oshima Y , Nanami T , Sumazaki M , et al. High serum PD‐L1 level is a poor prognostic biomarker in surgically treated esophageal cancer. Cancer Med. 2020;9:1321–7. 31865635 10.1002/cam4.2789 PMC7013049

[80] Takeuchi M , Doi T , Obayashi K , Hirai A , Yoneda K , Tanaka F , et al. Soluble PD‐L1 with PD‐1‐binding capacity exists in the plasma of patients with non‐small cell lung cancer. Immunol Lett. 2018;196:155‐60. 29366663 10.1016/j.imlet.2018.01.007

[81] Alix‐Panabieres C , Pantel K . Challenges in circulating tumour cell research. Nat Rev Cancer. 2014;14:623–31. 25154812 10.1038/nrc3820

